# Rapid habituation of the cold shock response

**DOI:** 10.1186/2046-7648-4-S1-A38

**Published:** 2015-09-14

**Authors:** Clare M Eglin, George Butt, Stephen Howden, Thomas Nash, Joseph Costello

**Affiliations:** 1 Extreme Environments Laboratory, Department of Sport and Exercise Science, University of Portsmouth, Portsmouth, UK

## Introduction

Sudden immersion into cold water initiates a series of cardio-respiratory responses collectively known as the cold shock response (CSR) which may increase an individual's risk of drowning. The CSR is stimulated by a rapid fall in skin temperature and includes tachycardia, a reflex inspiratory gasp followed by uncontrollable hyperventilation. Repeated cold water immersions conducted over several days have been shown to reduce the magnitude of the CSR [1]. This study investigated whether an habituation could be achieved in a couple of hours; it was hypothesised that following this rapid habituation the CSR would be reduced on a subsequent cold water immersion.

## Methods

Nine healthy males (mean [SD] age 21[2] years, height 179[7] cm, mass 76[13] kg) with no previous cold water exposure, undertook two head-out immersions into stirred water at 15 °C for 5 minutes wearing swimming trunks. These immersions were undertaken one week apart at the same time of day (IMM1 and IMM7). One or two days after IMM1, participants undertook five, 3-minute, head-out immersions into 15 °C water over a period of 55 to 120 min. In between each habituation immersion they rewarmed in a bath at 38 °C for 3 minutes and the next immersion occurred approximately 10 minutes later. Heart rate (f_C_), respiratory frequency (f_R_), tidal volume (V_T_) and inspiratory minute volume (V_I_) were measured prior to and during each immersion. Data for IMM1 and IMM7 were averaged over the following time periods: 0-30, 30-60, and 60-300 s and are presented as mean[sd].

## Results

f_C _was reduced throughout IMM7 compared to IMM1 (0-30 s: 117[21] v 106[14] bpm; 30-60 s: 110[21] v 86[19] bpm; 60-300 s: 90[18] v 78[17] bpm; all P < 0.05). V_I _was attenuated in IMM7 compared to IMM1 over the first minute of immersion (0-30 s: 61.3[7.5] v 52.5[12.1] L.min^-1^; 30-60s: 50.8[13.5] v 40.5[13.6] L.min^-1^; P < 0.05) whereas f_R _was only reduced in the first 30 s from 37(11) to 29(9) breaths.min^-1 ^(P < 0.05). The inspiratory gasp observed within the first 10 s of immersion was similar in IMM1 and IMM7 (2.44[0.62] v 2.71[0.64] L, p > 0.05) as was V_T _throughout the immersions.

## Discussion

Repeated immersions conducted over a short time period (1-2 hours) on the same day resulted in a decrease in f_C_, f_R _and V_I _during the first 30 s of immersion. This may reduce the risk of drowning by attenuating ventilation and thus the risk of water aspiration as well as lowering f_C _and therefore cardiac strain on immersion. No reduction in either V_T _or the inspiratory gasp was observed, probably because f_R _decreased giving a longer duration for each inspiration. As previous studies [2] have indicated that f_R _is a better indicator of respiratory drive than V_T _during the CSR, the current findings suggest that respiratory drive was reduced following the rapid habituation protocol.

## Conclusion

Rapid habituation to the CSR is possible and may provide a practical and inexpensive method of protection against drowning for individuals who are deployed at short notice to situations where they are at risk of accidental cold water immersion.

## References

[B1] Tipton Temperature dependence of habituation of the initial responses to cold water immersion Eur J Appl Physiol 1998 78 253 57 10.1007/s004210050416 9721005

[B2] Tipton Human initial responses to immersion in cold water at three temperatures and after hyperventilation J Appl Physiol 1991 70 317 322 2010387 10.1152/jappl.1991.70.1.317

